# Biology of Infection and Disease Pathogenesis to Guide RSV Vaccine Development

**DOI:** 10.3389/fimmu.2019.01675

**Published:** 2019-07-25

**Authors:** Seyhan Boyoglu-Barnum, Tatiana Chirkova, Larry J. Anderson

**Affiliations:** ^1^Vaccine Research Center, National Institutes of Health, Bethesda, MD, United States; ^2^Department of Pediatrics, Emory University and Children's Healthcare of Atlanta, Atlanta, GA, United States

**Keywords:** pathogenesis, RSV (respiratory syncytial virus), vaccine development, biology of infection, protective immunity

## Abstract

Respiratory syncytial virus (RSV) is a leading cause of severe lower respiratory tract disease in young children and a substantial contributor to respiratory tract disease throughout life and as such a high priority for vaccine development. However, after nearly 60 years of research no vaccine is yet available. The challenges to developing an RSV vaccine include the young age, 2-4 months of age, for the peak of disease, the enhanced RSV disease associated with the first RSV vaccine, formalin-inactivated RSV with an alum adjuvant (FI-RSV), and difficulty achieving protection as illustrated by repeat infections with disease that occur throughout life. Understanding the biology of infection and disease pathogenesis has and will continue to guide vaccine development. In this paper, we review the roles that RSV proteins play in the biology of infection and disease pathogenesis and the corresponding contribution to live attenuated and subunit RSV vaccines. Each of RSV's 11 proteins are in the design of one or more vaccines. The G protein's contribution to disease pathogenesis through altering host immune responses as well as its role in the biology of infection suggest it can make a unique contribution to an RSV vaccine, both live attenuated and subunit vaccines. One of G's potential unique contributions to a vaccine is the potential for anti-G immunity to have an anti-inflammatory effect independent of virus replication. Though an anti-viral effect is essential to an effective RSV vaccine, it is important to remember that the goal of a vaccine is to prevent disease. Thus, other effects of the infection, such as G's alteration of the host immune response may provide opportunities to induce responses that block this effect and improve an RSV vaccine. Keeping in mind the goal of a vaccine is to prevent disease and not virus replication may help identify new strategies for other vaccine challenges, such as improving influenza vaccines and developing HIV vaccines.

## Background

Respiratory syncytial virus (RSV) is estimated to cause 3.4 million hospitalizations and 95,000–150,000 deaths globally and up to 175,000 hospitalizations in the United States in children <5 years of age each year (1, 2). It is also estimated to cause 14,000 deaths each year in adults in the United States (3). Its disease burden has made RSV a priority for vaccine development for over 50 years but no vaccine is yet available for any of groups targeted for an RSV vaccine including young children (~ <6 months of age), older children (~ 6 months to 24 months of age), pregnant women, and elderly adults (~ >65 years of age) (4, 5). The challenges to developing an RSV vaccine include: concern that a non-live virus vaccine in young children may predispose to enhanced RSV disease (ERD) in RSV-infected young children who earlier received a formalin-inactivated RSV plus alum vaccine; difficulty in inducing and assessing protective immunity; cost of clinical vaccine trials; and the young age, 2–4 months of age, for peak of disease. The first RSV vaccine, formalin-inactivated RSV with alum adjuvant (FI-RSV), given to young, likely RSV naïve, but not older, RSV primed children, led to enhanced RSV disease (ERD) with later infection, i.e., a high rate of hospitalization and two deaths (6–9). This experience raised concern that any non-live virus vaccine may induce an aberrant immune response that predisposes to ERD in young children and a focus on live attenuated RSV or virus vector vaccines for this target population. Since ERD is not a concern for RSV-primed older children and adults and live attenuated RSV replicates poorly in primed persons, subunit vaccines are under development for older children and adults. The difficulty in inducing protective immunity is highlighted by repeat infections and disease throughout life (3, 10).

The fact that prior infection and high titers of neutralizing antibodies, e.g., maternally derived antibodies or from an earlier infection, are associated with some protection suggest that a vaccine should be achievable (11–17). In addition, immune globulin with a high RSV neutralizing antibody titer and a neutralizing monoclonal antibody are effective in preventing serious disease in high-risk young infants (18, 19).

The past failures, however, suggest that novel vaccines may be required for success. In considering novel vaccines, it is useful to remember that the goal of a vaccine is to prevent disease caused by the infection. Though obviously important to an effective vaccine, a singular focus on induction of neutralizing antibodies or preventing virus replication, may lead to missing other, important effects of a vaccine. For example, if a vaccine does not induce sterilizing immunity, as is likely for RSV, other effects such as virus-induced inflammation become relevant. The pathogenesis of RSV disease, reviewed elsewhere (20, 21), is the foundation for designing a vaccine that addresses disease pathogenesis. The prominence of wheezing as a manifestation of infection (10) with its similarity to asthma and the association between mucus production and disease severity (22) suggest a prominent role of host inflammatory responses in disease pathogenesis. Blocking such effects could be important to a successful vaccine.

The role of RSV's proteins in biology of infection and disease pathogenesis provides clues to their potential contribution to a vaccine. RSV has 10 genes that encode for 11 proteins (23). RSV has two major antigenic groups of strains, A and B, and multiple genotypes within the two groups (24–27). Though only two RSV proteins induce *in vitro* neutralizing antibodies, F and G (28), as illustrated in Table 1, all RSV proteins have played a role in design of one or more vaccines. The type of vaccine under development varies among the target populations. Live attenuated or virus-vector subunit vaccines are under development for infants and young children and non-live or virus-vector subunit vaccines for older children and adults.

**Table 1:** RSV proteins in live attenuated or subunit vaccines.

**Protein**	**Size aa**	**Functions related to vaccine design**	**Role in a live virus vaccine**	**Role in a subunit vaccine**
NS1	139 aa	Inhibits type 1 interferon production to block host response to control infection	Attenuation when deleted or codon de-optimized	None
NS2	124 aa	Inhibits type 1 interferon production to block host response to control infection	Attenuation when deleted or codon de-optimized	None
Nucleoprotein (N)	391 aa	Nucleocapsid formation and T cell epitopes	Attenuation or temperature sensitivity when mutated	Induce T cell immunity
Phosphoprotein (P)	241 aa	Nucleocapsid formation, replication	Attenuation when codon pair de-optimized	Platform for RSV VLPs
Matrix protein (M)	256 aa	Envelop, virion assembly	Attenuation and temperature sensitivity when the gene start signal mutated	Induce T cell immunity and platform for RSV VLPs
Small hydrophobic (SH)	64 aa	Ion channel	Attenuation when deleted or codon pair de-optimized	Induce ADCC antibodies to decrease virus replication
G protein	292-319 aa	Attachment and immune modulation	Attenuation when deleted and improved safety and immunogenicity when mutated	Induce antibodies to inhibit virus replication by blocking binding to the cell surface receptors CX3CR1 and glycosaminoglycans and/or ADCC and to block virus-induced inflammation
F protein	574 aa	Attachment, entry, fusion	Attenuation when mutated or codon pair de-optimized and improved protective immunity and virus stability when mutated	Induce antibodies to inhibit virus replication by blocking fusion and possibly by ADCC
M2-1 protein	194 aa	Anti-termination factor during transcription	Attenuation when mutated	Induce T cell immunity, platform for RSV VLPs
M2-2 protein	90 aa	Switch from transcription to replication	Attenuation and enhanced immunity when deleted	None
L protein	2,165 aa	Viral polymerase	Attenuation when mutated or codon pair de-optimized	None

*Adapted with permission from Anderson (29)*.

## Live Virus Vaccines

A live attenuated RSV vaccine needs to both have mutations that attenuate virus replication for safety while maintaining sufficient replication to maintain immunogenicity. The first attenuated vaccines were generated by chemical mutagenesis and low temperature passage. Subsequently, reverse genetics has identified specific mutations associated with temperature sensitivity and attenuation (30, 31). A set of five mutations, one in the N, two in the F, and two in the L protein genes, are associated with attenuation in primates and designated “cp” for cold passage. Six additional mutations, 5 in L and 1 in the gene-start transcription signal for M2, contribute independently to temperature sensitivity and attenuation. Five RSV genes, i.e., NS1, NS2, SH, G, and M2-2, can be deleted and virus recovered. All viruses are attenuated in animals. Live attenuated RSV candidate vaccines with deletions of NS2, G, or M2-2 are in clinical trials (32). A live attenuated RSV candidate vaccine with the 5 cp mutations, two other attenuating mutations, and deletion of the SH gene was also in a clinical trial (33, 34).

A virus vector vaccine's safety is likely not dependent on the RSV antigen present but the vector. Since the virus vectors present antigen to the immune system similar to the way that live RSV does, they are likely safe from ERD risk. A parainfluenza virus that expressed the RSV F protein did not led to ERD in RSV naïve children (35).

Codon pair de-optimization is another way to attenuate RSV and different combinations of RSV proteins including NS1 and NS2; NS1, NS2, N, P, M, and SH; G and F; L; or all proteins except M2-1 and M2-2 have been codon de-optimized to attenuate the virus (36, 37). With codon pair de-optimization, the level of attenuation can be fine-tuned by varying levels of protein production and makes it possible to attenuate through changes to any protein without relying on specific attenuating mutations or gene deletion.

Several live attenuated RSV vaccines show promise in early clinical trials (38). It is yet uncertain if they will achieve the balance between safety and immunogenicity needed for the young child. Maternal vaccination, or longer lasting immune prophylaxis, followed by vaccination at 4–6 months of age should make safety easier to achieve. A safe virus vector is another possible way to protect young children.

## Subunit Vaccines

With the exception of virus-vector subunit vaccines, subunit vaccines are under development for RSV-primed older children and adults. Virus vector vaccines are under development for both. The goal for a subunit vaccine is to safely, induce a more effective immune response than natural infection. One or both of RSV proteins that induce neutralizing antibodies (F and G) are likely required for an effective subunit vaccine. Proteins that induce T cell immunity (N, M2-1, and other proteins) or antibody dependent cellular cytotoxic antibodies (ADCC) including the F, G, and SH proteins are incorporated into subunit vaccines (Table 1). Co-expression of the M protein and P proteins produces RSV virus-like-particle (VLPs) vaccine platform. A number of subunit vaccines, some in a virus vectors, including F protein; G protein; SH protein; F plus G; or F, G, and other RSV proteins are under study in clinical trials (4, 32).

Several pre-fusion F subunit vaccines are in early clinical trials and expected to induce higher titers of neutralizing antibodies and be more effective than previous F protein vaccines. Recently, two non-prefusion stabilized F protein vaccines were ineffective in elderly adults in phase II or III clinical trials (4). In a phase III maternal vaccination trial, one of these F protein vaccines did not significantly decrease medically significant RSV lower respiratory tract illness in infants (its primary endpoint) but did significantly decrease hospitalization in the infant (38), a result that suggests an F protein can be an effective maternal vaccine. An extended half-life, anti-F neutralizing monoclonal antibody is in phase II or III clinical trials and a promising alternative to vaccination to protect infants (39).

## Function and Role of RSV Proteins in Vaccine Design

As noted above and outlined in Table 1, all RSV proteins are included in design of one or more vaccines. Understanding the role of RSV proteins in the biology of infection and disease pathogenesis helps determine if, and how, individual proteins might contribute to a vaccine.

Below we discuss each proteins function relative to vaccine design with an emphasis on the F and G proteins. F and G are most effective at inducing protective immunity and one or both likely needs to be included in a RSV vaccine. Though G is often not included in candidate vaccines, its role in disease pathogenesis suggest it might make important contributions to a vaccine.

### NS1 and NS2 Proteins

NS1 is a 139 aa and NS2 is a 124 aa non-structural proteins, i.e., not incorporated into the virus but produced during transcription and replication. They both participate in virus replication and antagonize host innate responses designed to control infection (40–49). Deleting or codon de-optimizing he gene ability to alter host cell responses that control the infection that reduces virus replication and attenuates the virus (36, 37, 50–55).

### N Protein

The 391-amino acid N protein binds to and encapsidates the viral RNA generating an RNAse resistant nucleocapsid that is the template for transcription and replication of RSV genome (56, 57). N also inhibits host cell down regulation of cellular and viral protein production (58) and may impair dendritic cell and T cell interactions (59). It does not induce neutralizing antibodies but does induce T cell responses that protect animals at 4 weeks post vaccination (28, 60, 61). Given its role in virus replication, de-optimizing N gene codons should attenuate a live virus vaccine and its induction of T cell responses might contribute to efficacy of subunit vaccines.

### P Protein

The P protein is a 214 aa protein that is part of the ribonucleoprotein complex (RNP) (56, 57). The P protein interacts with both the N and L proteins and is an essential co-factor for L function. P also interacts with the M2-1 protein (62). Since co-transfection of P and M proteins produces RSV VLPs (63), it could be used in a subunit RSV VLP vaccine. P's role in virus replication suggest that de-optimizing P gene codons should attenuate the virus (36).

### M Protein

The M protein is 256 aa and guides assembly, budding, and virion formation (64). It lines the inner surface of the viral envelop, helps determine the shape of virus particles, and, with P, forms VLPs (63, 65–69). Since M induces T cell responses in vaccinated animals and memory T cells in humans after natural infection (70, 71), it might improve a subunit vaccines efficacy.

### SH Protein

The SH, small hydrophobic protein is a 64–65 amino acid type II protein located on the surface of the virus. It forms a pentameric cation-selective ion channel, or a viroporin, and can activate NLRP3 inflammasome leading to IL-1b expression (72, 73). Deletion of the SH gene is often used to attenuate live RSV candidate vaccine strains (74). Codon pair de-optimization (CPD) (36, 75) might also attenuate the virus. Though SH does not induce neutralizing (76), an SH vaccine induces antibody-dependent cell-mediated cytotoxicity (ADCC) antibodies and protection in animals (77, 78) and being studied in clinical trials (4, 32).

### G Protein

The G protein is a class II protein of 292-319 amino acids (AA) long. The extracellular domain contains a variable, highly glycosylated domain and a central conserved domain (CCD-G) followed by a second variable, highly glycosylated domain. Within the CCD-G are 13 aa conserved among all strains (aa 164-176) and a CX3C chemokine motif (aa 182-186). Through the CX3C motif, G, like the one CX3C chemokine, fractalkine, binds to the chemokine receptor CX3CR1 (79). G, as does F, also binds to cell surface glycosaminoglycans (GAGs) through its heparin binding domains and GAGs are one receptor for RSV infection. In primary human airway epithelial cells, RSV also uses CX3CR1, through the CX3C motif in G, as a receptor for infection (80–82). G binding to CX3CR1 can also induce fractalkine-like responses (79). CX3CR1 is expressed on the surface of many cell types, including neurons and microglia (83), smooth muscle (84), and various immune cells including monocytes, dendritic, NK, T, and B cells (85–87) and binding to it can induce a variety of downstream responses. In mice, the G protein/CX3CR1 interaction is associated with depressed respiratory rates (88), inhibition of migration of CX3CR1+ T cells to RSV-infected lungs (89), induction of aberrant pulmonary inflammation with RSV challenge after FI-RSV vaccination (90), increased pulmonary inflammation and mucous production and airway resistance during infection, and induction of Th2-type immune responses in the lung with infection (91). In *In vitro* studies, the G protein through its interaction with CX3CR1 dampens Type I IFN production by innate immune cells and Type 1 cytokine responses of memory T cells (92). Recently, the G-CX3CR1 interaction has been shown to induce IL-10 in neonatal regulatory B cells (nBreg) resulting in downregulation of Th1 cell responses (93).

**Figure 1:**
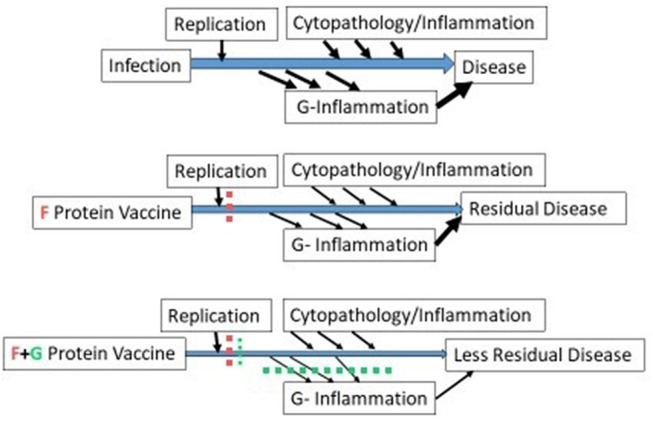
Enhanced disease prevention with the addition of G to an F protein vaccine. The three schematics represent disease pathogenesis associated with no vaccine (1st schematic), an F protein vaccine (2nd schematic), and an F + G protein vaccine (3rd schematic). For all three, two types of disease pathogenesis are represented, one associated with virus replication and cytopathology (above the line) and the other induced by the RSV G protein (below the line). In mice, G induced disease includes increased inflammatory cells and mucus in the lungs and increased signs of obstructive airway disease and is not dependent on level of virus replication (95–97). In the second schematic, an F protein vaccine prevents much but not all virus replication and much of the disease pathogenesis represented above the line. In the third schematic, addition of G to an F protein also prevents disease pathogenesis represented below the line. The width of the arrows indicate level of virus replication, cytopathology/inflammation, G-inflammation, or residual disease.

The ability of the anti-G monoclonal antibody, 131-2G, to block these effects of G (91, 94–97) suggests a role for G in vaccine design. As illustrated in Figure 1, immunity designed to block infection, if successful, will prevent disease. However, if only partially successful, as occurs with naturally acquired immunity, RSV will replicate and produce G leading to G induced host immune/inflammatory responses that cause disease. Vaccine-induced anti-G antibodies can block G-induced disease and essentially have an anti-inflammatory effect that decreases disease. Interestingly, the anti-inflammatory effect of 131-2G is independent of its anti-viral effect, i.e., intact 131-2G has both an anti-viral effect and anti-inflammatory effect while 131-2G F(ab')2 has no anti-viral effect but a similar anti-inflammatory effect (95, 96). Since CX3CR1 is an important receptor in primary human airway epithelial cells, likely in natural human infection, antibodies that block G's interaction with CX3CR1 should neutralize virus in humans by a mechanism different from F. Finally, studies in mice suggest that anti-G immunity, through passively administered 131-2G before RSV challenge or actively induced by a CCD-G peptide vaccine given with FI-RSV, can block ERD in RSV-challenge of FI-RSV vaccinated mice (98, 99). These data suggest that including G, or a CCD-G containing peptide, in an RSV vaccine might decrease the risk of ERD in infants and young children.

Thus, G in a subunit vaccine can induce antibodies that block binding to CX3CR1 that should enhance the antiviral activity of an F protein subunit vaccine and uniquely add an anti-inflammatory effect not present in an F only vaccine (Figure 1). In a live attenuated vaccine, mutating G to block binding to CX3CR1, from studies in mice, should markedly decreased disease and maintain, or enhance, the vaccine-induced immunity (100).This mutation by blocking binding to CX3CR1 would also attenuate virus replication in humans.

Thus, including G in RSV vaccine design could improve a vaccine through multiple mechanisms. A number of G, or G peptides that include CCD-G, vaccines have been effective in preventing disease in animal studies (101–109). A G construct based on CCD-G will likely need to account for antigenic differences between groups A and B and not within the two groups.

### F Protein

The F protein is a class I fusion protein of 574 amino acids (AA) long. It has two furin cleavages sites, at aa position 109 and the other at aa 136. Cleavage at these sites gives the 50 kDa carboxy-terminal F1, the 20 kDa N-terminal F2, and a 27 aa fragment. F1 and F2 form dimers and the F1-F2 dimers form trimmers (110, 111). The F protein is highly conserved among RSV strains with 25 AA differences between RSV subtypes A and B and induces neutralizing antibodies and protection in animals across the two groups (110, 112). F binds to glycosoaminoglycans (113), nucleolin (114), and EGFR (115) on the cell surface with GAGs and nucleolin presumed to be receptors for infection of cells. F binding to EGFR is associated with induction of IL-13 and mucin production.

The F protein mediates fusion of RSV with cellular membranes which is essential to infection and requires F to go from the metastable pre-fusion (pre-F) structure to a stable post-fusion (post-F) structure (116, 117). Many neutralizing epitopes on F are on the pre- and not post-fusion structure and most of the neutralizing antibodies in humans react against the pre- and not post-fusion form of F (118, 119). Pre-fusion stabilized F protein constructs have been developed and these F constructs, e.g., Ds-Cav1 and SC-TM, are highly effective at inducing neutralizing antibodies (120). Anti-F antibodies can also mediate antibody dependent cell-mediated cytotoxicity (ADCC) (121, 122) though it is unknown what role ADCC antibodies play in controlling natural infection. The initial two neutralizing antigenic sites identified on F have been expanded to at least five and more will likely be identified in the future (24, 123). Anti-antigenic site Ø antibodies have high levels of neutralizing activity and are a high proportion of neutralizing antibodies in human serum specimens (118). Interesting, F proteins in some circulating strains have been shown to have increased stability of pre-fusion F, increased virus temperature stability, induce mucus and airway resistance in mice, and bind to EGFR (115, 124, 125).

Stabilization of pre-fusion F in subunit vaccines substantially increases the neutralizing antibody response in animals and is a promising development in design of RSV subunit vaccines. In a live virus vaccine, mutations in F that increase pre-fusion stability and temperature stability should be advantageous. Mutations at other sites in F have been associated with virus attenuation. It is possible that mutations that block F binding to EGFR will attenuate disease and improve a live attenuated RSV vaccine.

The F protein's essential role in infection through fusion suggest it is key to protection for both subunit and live virus vaccines.

### M2-1 and M2-2 Proteins

The internal viral matrix protein M2 is unique to the family Pneumoviridae, plays a significant role in virus assembly (66), and contains two overlapping translational open reading frames, one for M2-1, a 194 aa protein, and one for M2-2, a 83-90aa protein (126).

The *M2-1* protein functions as an intragenic transcription anti-termination factor allowing the synthesis of complete RNA (127–129) and link the RNA/nucleocapsid with the M protein just inside the virus surface (67, 130). M2-1 can induce short term, T cell based RSV immunity (28, 70, 131) and could be included in a subunit vaccine to enhance induction of T cell immunity.

The *M2-2* protein facilitates the shift from gene transcription to production of viral RNA and infectious virus (126, 132). Deletion of M2-2 results in a decrease genome replication and increase in gene transcription and protein production resulting in both attenuation and increased immunogenicity. M2-2 deletion viruses are being evaluated in a phase 1 clinical trials (34, 133).

### L Protein

The L protein is a large, 2,165-amino acid, protein that mediates transcription and replication of RSV RNA and capping and methylation of mRNA (56, 57, 134). The active form of L is a heterodimer of the L and P proteins with P essential to L's catalytic activity. Given its central role in transcription and replication it is not surprising that attenuating mutations, and likely codon pair de-optimization of L, attenuate live RSV (30, 31).

## Comment

Though a number of candidate RSV vaccines are under development and some promising candidate vaccines have moved into clinical trials, past failures suggest that we should continue look for better candidate vaccines. Though the composition of a successful RSV vaccine remains uncertain, it likely will need to induce both antibody and Th1 biased T cell memory responses. It is, also, useful to remember that the goal is to prevent disease and not just to control infection. For example, tetanus and diphtheria toxoid vaccines prevent the disease and not pathogen growth. The RSV G protein has the potential to enhance a vaccine by not only helping to control infection but independently decreasing disease by controlling virus-induced inflammation. Virus protein-specific contributions to biology of infection and disease pathogenesis might also suggest ways to decrease disease for other vaccine challenges such as improving influenza vaccines and developing HIV vaccines.

## Author's Note

Respiratory syncytial virus is a high priority for vaccine development but, despite nearly 60 years of research no vaccine is yet available. Understanding the biology of infection and pathogenesis of disease has and will continue to be key to developing new vaccine strategies to finally achieve a successful vaccine. New vaccines are being developed and their safety and efficacy will ultimately be determined by clinical trials in the target population. Given past failures it is important to continue to pursue better candidate vaccines. In developing new vaccines, it is useful to remember that the goal of a vaccine is to prevent disease and not, though essential to an effective vaccine, virus replication.

## Author Contributions

All authors listed have made a substantial, direct and intellectual contribution to the work, and approved it for publication.

### Conflict of Interest Statement

LA has done paid consultancies on RSV vaccines for Moderna Therapeutics, Inc., Bavarian Nordic, Novavax, Daiichi-Sankyo, and ClearPath Vaccines Company. LA's laboratory is receiving funding through Emory University from Pfizer for RSV surveillance studies in adults. LA is a co-inventor on several CDC patents on the RSV G protein and its CX3C Q10 chemokine motif relative to immune therapy and vaccine development. LA is also a co-inventor on an Emory patent filing for use of RSV platform VLPs with the F and G proteins for vaccines. The remaining authors declare that the research was conducted in the absence of any commercial or financial relationships that could be construed as a potential conflict of interest.
